# Mechanical stress regulates autophagic flux to affect apoptosis after spinal cord injury

**DOI:** 10.1111/jcmm.15863

**Published:** 2020-09-17

**Authors:** Xin Zhang, Yingli Jing, Chuan Qin, Changbin Liu, Degang Yang, Feng Gao, Mingliang Yang, Liangjie Du, Jianjun Li

**Affiliations:** ^1^ School of Rehabilitation Medicine Capital Medical University Beijing China; ^2^ China Rehabilitation Science Institute Beijing China; ^3^ Center of Neural Injury and Repair Beijing Institute for Brain Disorders Beijing China; ^4^ Department of Spinal and Neural Functional Reconstruction China Rehabilitation Research Center Beijing China; ^5^ Beijing Key Laboratory of Neural Injury and Rehabilitation Beijing China; ^6^ Institute of Rehabilitation medicine China Rehabilitation Research Center Beijing China; ^7^ Department of Rehabilitation Medicine Beijing Tiantan Hospital Beijing China

**Keywords:** apoptosis, autophagic flux, decompression, mechanical stress, mTOR, spinal cord injury

## Abstract

Increased mechanical stress after spinal cord injury (SCI) expands the scope of nerve tissue damage and exacerbates nerve function defects. Surgical decompression after SCI is a conventional therapeutic strategy and has been proven to have neuroprotective effects. However, the mechanisms of the interaction between mechanical stress and neurons are currently unknown. In this study, we monitored intramedullary pressure (IMP) and investigated the therapeutic benefit of decompression (including durotomy and piotomy) after injury and its underlying mechanisms in SCI. We found that decreased IMP promotes the generation and degradation of LC3 II, promotes the degradation of p62 and enhances autophagic flux to alleviate apoptosis. The lysosomal dysfunction was reduced after decompression. Piotomy was better than durotomy for the histological repair of spinal cord tissue after SCI. However, the autophagy‐lysosomal pathway inhibitor chloroquine (CQ) partially reversed the apoptosis inhibition caused by piotomy after SCI, and the structural damage was also aggravated after CQ administration. An antibody microarray analysis showed that decompression may reverse the up‐regulated abundance of p‐PI3K, p‐AKT and p‐mTOR caused by SCI. Our findings may contribute to a better understanding of the mechanism of decompression and the effects of mechanical stress on autophagy after SCI.

## 1 INTRODUCTION

Spinal cord injury (SCI) is a serious health problem worldwide with a heavy burden,^1^ but there is currently no effective clinical treatment strategy. The pathology of and repair mechanisms for SCI are mainly focused on the prevention and reversible regulation of secondary injury, preventing further death of neurons in the spinal cord, enhancing neural plasticity and promoting nerve repair.^2^

Spinal cord injury not only involves a brief primary injury but also includes delayed and persistent tissue damage, namely, a secondary injury, following the primary injury.^3^, ^4^ After SCI, spinal cord oedema or bleeding^5^, ^6^ coupled with spinal canal compression and inhibition of the dura mater increases intramedullary pressure (IMP).^7^, ^8^ This series of secondary injury events expands the range of neural tissue damage and exacerbates neurological deficits.^9^, ^10^ Changes in mechanical stress after SCI expand the scope of nerve tissue damage and exacerbate nerve function defects. A growing number of studies support early surgical decompression of traumatic SCI.^11^, ^12^, ^13^, ^14^, ^15^ However, the mechanisms by which mechanical stress affects neurorepair are not clear at present.

In vitro studies have shown that physiological mechanical stress induces autophagy and is independent of classical TOR/Akt or AMPK signalling.^16^ Trabecular meshwork cells activate autophagy in a mTOR‐ and BAG3‐independent manner under biaxial mechanical stretch and high pressure, thereby maintaining cell homoeostasis and adapting to mechanical stress.^17^ Autophagy regulation may be a potential therapeutic strategy for SCI.^18^, ^19^, ^20^, ^21^ However, the effects of excessive mechanical stress in the spinal cord on autophagy and the effect of decompression are unknown after SCI.

The purpose of this study was to observe the histopathological effects of mechanical stress on the spinal cord in a rabbit model after SCI and to explore the mechanism of decompression from the perspective of autophagy. This study may have important implications for future attempts to apply pressure and autophagy as a treatment strategy for SCI.

## 2 MATERIALS AND METHODS

### 2.1 Animals and ethics statement

The healthy female Japanese White Rabbits at 2.5‐3.0 months old and 2.0‐2.5 kg body weight used in the study were purchased from the Beijing Jinmuyang Experimental Animal Breeding Co., Ltd., Beijing, China. All animal experiment protocols were reviewed and approved by the Animal Ethics Committee of Capital Medical University (AEEI‐2018‐008), and experimental animal welfare and ethical principles were followed. The animals were freely provided with standard feed and drinking water.

### 2.2 Spinal cord injury and decompression

**Figure 1:**
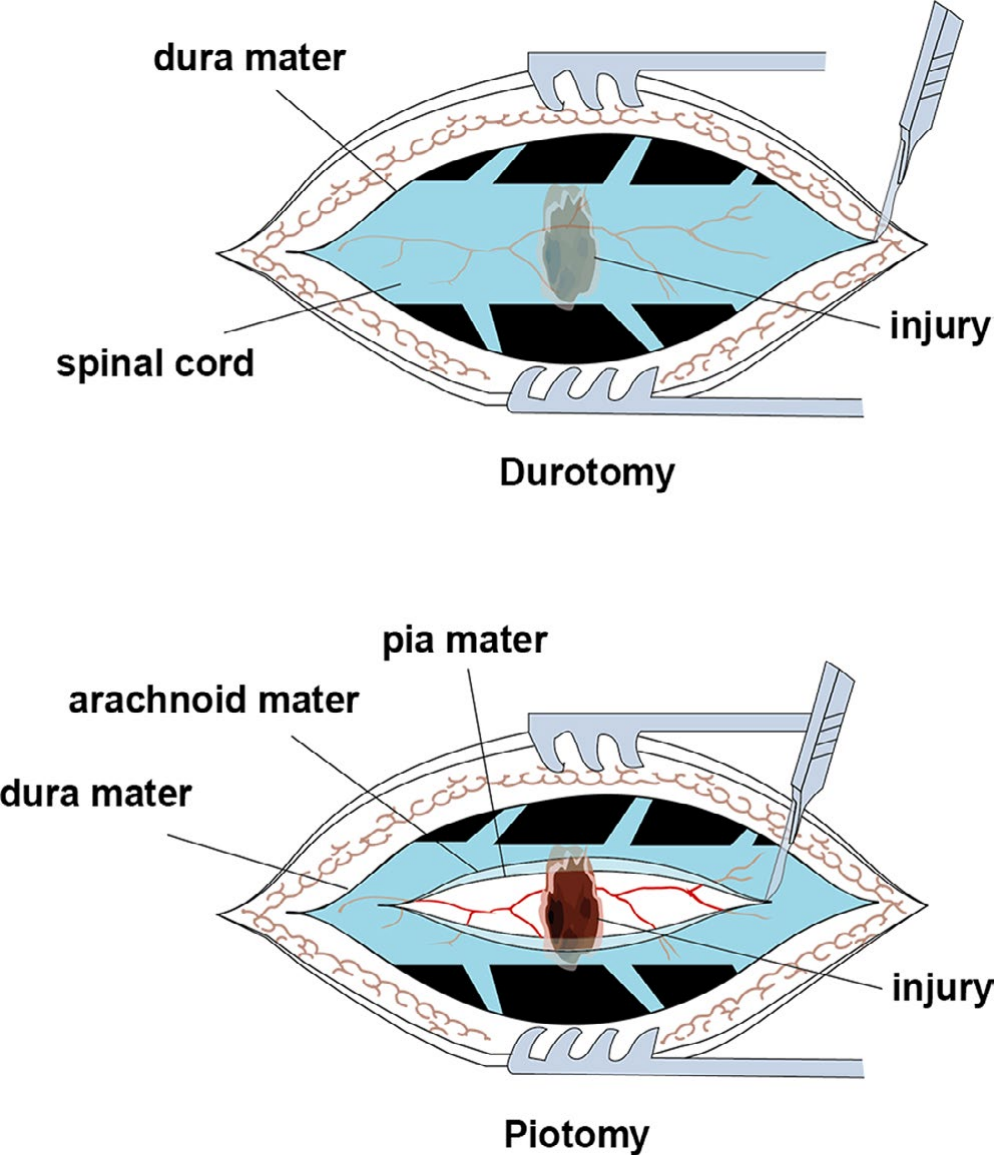
Illustration of durotomy and piotomy

The animals were fasted 12 hours before surgery, and sodium pentobarbital (3%, 30 mg/kg) was injected into the vein of the ear as anaesthesia. The rabbits were fixed on the operating table in a prone position and sequentially subjected to hair removal and disinfection. The processus spinosus and lamina of T9‐T11 were removed with a clamp to expose the spinal canal and spinal dura. The rabbits in the sham group only received laminotomy. The rabbits in the SCI group received spinal contusions using an aneurysm clip (REBSTOCK) with an intensity of 90 g and a retention time of 1 minute, and tail‐spasm swings and strong lower limb contractions were observed, thus resulting in SCI of T10. After SCI, only the dura mater was incised in the animals in the Durotomy group, keeping the arachnoid and pia mater intact. The rabbits in the Piotomy group received decompression after SCI by an approximately 2‐mm incision in the dura mater, arachnoid and pia mater incision along the midline of the spinal cord made with an 11‐point scalpel (Figure 1). After the completion of the experiment, the incision wound was washed with saline, sutured and sterilized. The cavity created by laminectomy was filled with a gelatin sponge (Gelfoam (Upjohn)), and the muscles, fascia and skin were sutured. Sodium lactate Ringer's injection (Shandong Qidu Pharmaceutical Co., Ltd.) was injected into the marginal vein during the operation until the animal fully recovered. The rabbits in the Piotomy + CQ group received chloroquine diphosphate salt (CQ) after Piotomy to inhibit autophagy and observe the effect of decompression on autophagic substrates degradation. CQ (c6628, Sigma‐Aldrich) was dissolved in 150 mg/mL physiological saline and administered intraperitoneally at 60 mg/kg/d.^22^ Subcutaneous injections of penicillin sodium (North China Pharmaceutical Co., Ltd.) and gabapentin (Hainan Selection Pharmaceutical Co., Ltd.) were administered post‐operatively for 3 days. The lower abdomen was massaged with a gentle squeezing of the bladder 3‐4 times each day to help with urination and defecation. All surgeries were performed by a surgeon under a microscope (OPMI 177736, Opton Co., Ltd.). After SCI, all rabbits were assigned to a treatment group according to a randomized block experimental design.

### 2.3 Locomotion recovery evaluation

Animals were removed from their cages and placed in an open field at 1, 2 and 3 days after SCI (n = 5 per group). Motor function of the hindlimbs was recorded according to the modified Tarlov scale (score 0‐5).^23^ Each animal was observed by two evaluators who were blinded to the treatment.

### 2.4 IMP monitoring

Intramedullary pressures were measured using Millar Telemetry Systems (MKT0002/D; Millar).^24^ After SCI (or decompression), the IMP was recorded at 1, 10, 24, 36 and 72 hours (n = 5 per group). All animals were continuously monitored for 1 hour at the corresponding time points, and the results were averaged.

### 2.5 Haematoxylin‐eosin (H&E) and Nissl staining

Fixed spinal cord tissue was subjected to dehydration, dipping in wax, embedding and sectioning at a thickness of 4 μm in preparation for parallel H&E and Nissl staining (n = 5 per group). Continuous coronal sectioning was performed. The ratio of the cavity area (cavity area/spinal cord cross‐sectional area) in the spinal cord was evaluated by H&E staining. The Nissl‐positive cells in the anterior horns of the spinal cord were counted (positive definition: intact cells, nuclei and axons) in a double‐blind manner, and the counts were averaged. All stained images were scanned at high resolution with HistoFAXS 3.0 (TissueGnostics), and the pictures were viewed using the associated professional viewing software (FAXS viewer).

### 2.6 Immunofluorescence

The wax‐embedded spinal tissue in each group (n = 5) was sectioned into slices at a thickness of 4 μm. After wax removal, hydration and antigen repair, the section was rinsed with phosphate‐buffered saline (PBS, pH = 7.4) three times for 5 minutes each time. Then, the section was subjected to blocking using 10% bovine serum albumin (BSA) for 30 minutes. The primary antibodies anti‐NeuN (MAB377; Millipore) and anti‐myelin basic protein (anti‐MBP, ab62631, Abcam) were added and incubated overnight at 4°C. After washing with PBS three times for 5 minutes each time, the secondary antibody (goat anti‐mouse, GB21301; goat anti‐mouse, GB25301; Servicebio) was incubated at room temperature for 50 minutes. Then, DAPI staining, antiquenching and sealing were conducted. The fluorescence images were scanned with a Pannoramic 250 (Pannoramic MIDI; 3D HISTECH); then, the images were viewed with CaseViewer (3D HISTECH) software. Photoshop CC (Adobe) and Image‐Pro Plus 6.0 software (Media Cybernetics) were used to process and analyse the images. The integrated optical density (IOD) of MBP in the whole cross‐sectional image was quantified, the NeuN‐positive cells in the anterior horns of the spinal cord were manually counted, and the counts were averaged. Both tests were quantified in a double‐blind manner.

### 2.7 Western blot (WB)

Tissue homogenate protein extracts were used for WB detection (n = 3 per group), and the expression of proteins was analysed. Animal tissues were lysed with RIPA buffer. Tissue homogenates were centrifuged at 9600 g for 10 minutes at 4°C. The extracted supernatant was quantified by the BCA assay. The proteins in the samples were subjected to SDS‐PAGE, transferred to a PVDF membrane, blocked with 5% skim milk and subjected to TBST washing three times for 10 minutes each time. Then, probing was performed with primary antibodies against Bcl‐2 (12789‐1‐AP; Proteintech), Bax (50599‐2‐Ig; Proteintech), p‐AKT (phospho‐PRAS40, Thr246, #2997, Cell Signaling Technology), p‐mTOR (phospho S2448, ab84400, Abcam), p‐PI3K (phospho Y607, ab182651, Abcam), p‐ULK1 (Ser757, #6888, Cell Signaling Technology), LC3 (#2775, Cell Signaling Technology), p62 (LS‐C313090, LifeSpan Biosciences), Cathepsin D (CTSD, ab75852, Abcam), LAMP1 (#9091, Cell Signaling Technology) with incubation overnight at 4°C. Secondary antibodies against mouse IgG (HRP, #7076, Cell Signaling Technology) and rabbit IgG (HRP, #7074, Cell Signaling Technology) were then added, and the membrane was incubated with shaking for 60 minutes at 37°C, developed in a dark room and scanned after fixation. The band grey values were quantified by Image‐Pro Plus 6.0 (Media Cybernetics) and expressed as ratios to the values for β‐actin (ab8226, Abcam). Full Materials and Methods please see the Appendix S1.

## 3 RESULTS

### 3.1 Decompression reduced IMP, with the most significant decrease in the Piotomy group

**Figure 2:**
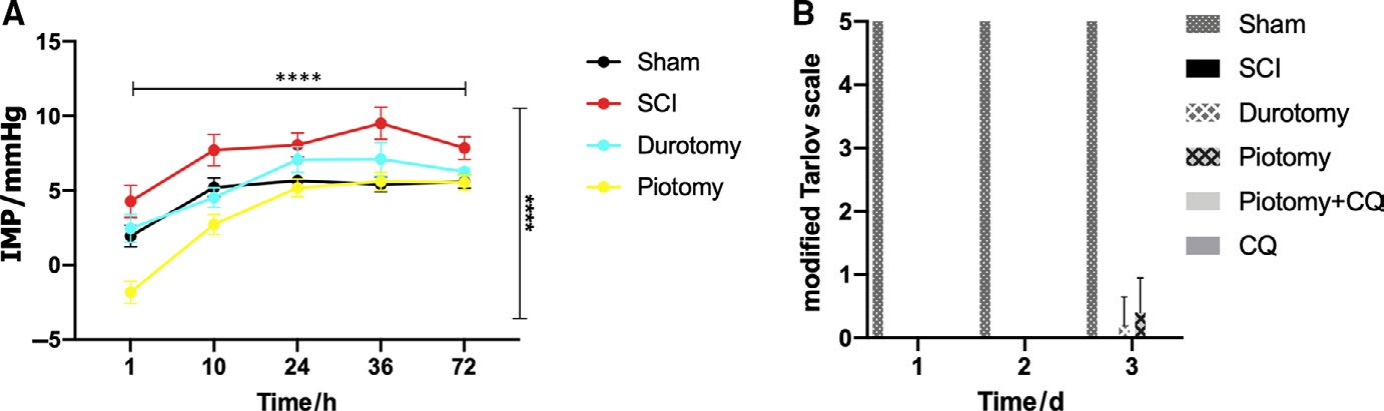
IMP and locomotion recovery evaluation in different groups. A, Decompression significantly decreased IMP after SCI. The IMPs were monitored in each group. The data are presented as the means ± SDs, n = 5 (the ‘n’ in the whole text refers to the number of samples (rabbits) in each group). Two‐way repeated measures ANOVA followed by Tukey's multiple comparisons test. ****P < .0001. The horizontal capped line suggests a significant effect of time on these parameters. The vertical capped line indicates the significant impact of group on them. B, Motor function of the hindlimbs was recorded according to the modified Tarlov scale. The data are presented as the means ± SDs, n = 5. Two‐way repeated measures ANOVA followed by Tukey's multiple comparisons test. SCI, spinal cord injury; CQ, chloroquine; IMP, intramedullary pressure

To observe the impact of decompression on IMP after SCI, we monitored the IMP at 1, 10, 24, 36 and 72 hours in the Sham, SCI, Durotomy and Piotomy groups. Compared with the Sham group, the SCI group exhibited a significantly increased IMP. Compared with the SCI group, the IMPs were decreased in the Durotomy and Piotomy groups, with the most significant decrease in the Piotomy group (Figure 2A).

### 3.2 Effects of decompression on neurologic outcome in the rabbit SCI model

To observe the locomotion recovery of rabbits, the motor function was evaluated by the modified Tarlov scale (Figure 2B). The rabbits in the Sham group showed no neurologic deficits. The Tarlov scores were 0 in the SCI group. Compared with SCI group, the Durotomy and Piotomy groups showed an increasing trend at 3 days after injury, but no significant difference was observed. Compared with the SCI group, no significant differences in the Tarlov scores were observed in the Piotomy + CQ and CQ group.

### 3.3 Decompression restored autophagic flux after SCI, and autophagic flux was impaired after CQ administration

**Figure 3:**
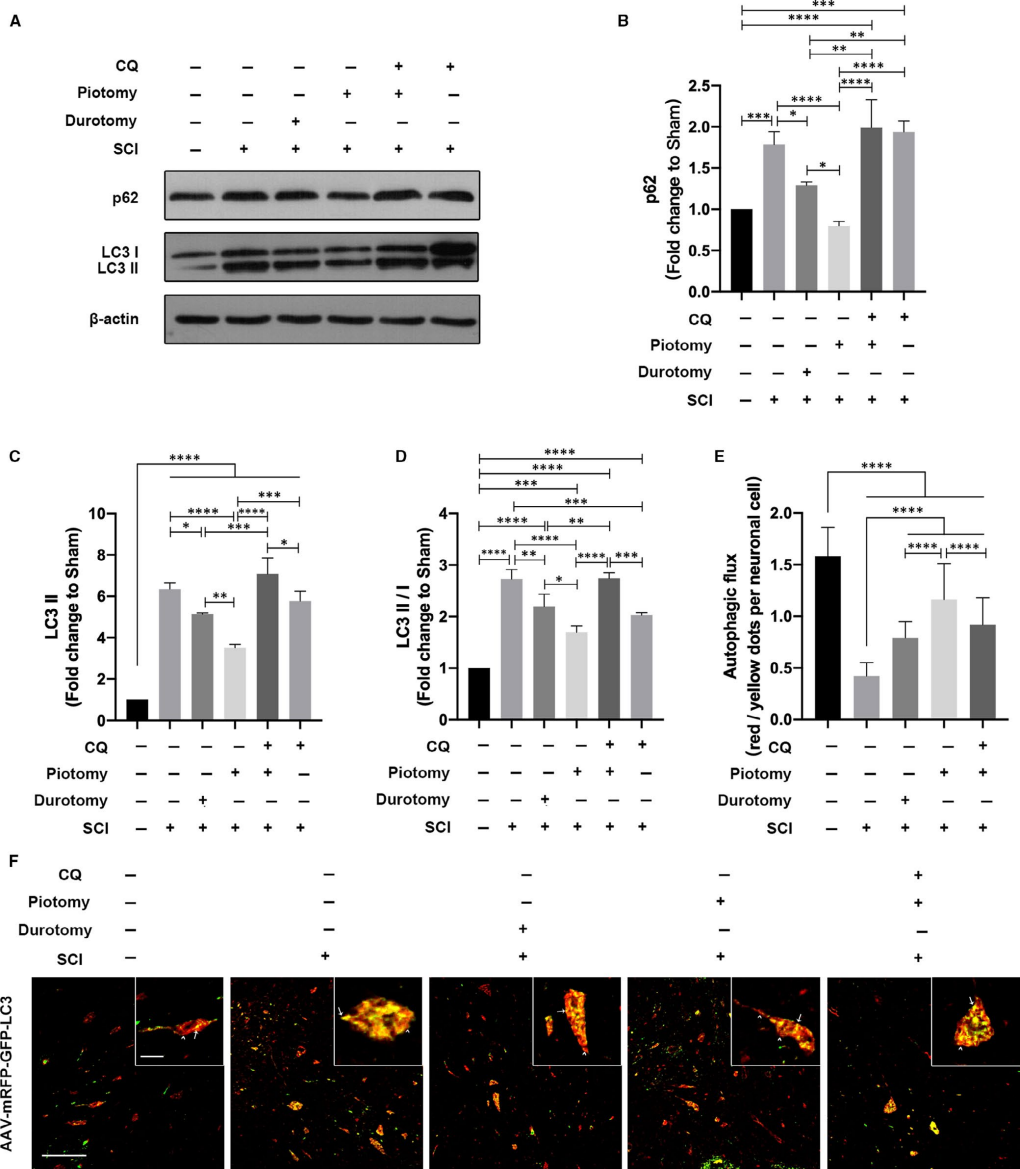
Decompression restored autophagic flux after SCI, whereas autophagic flux was impaired after CQ administration. A‐D, Western blotting and quantitative analysis of changes in p62 and LC3 expression in rabbits. The data are presented as the means ± SDs, n = 3. One‐way ANOVA followed by Tukey's multiple comparisons test. *P < .05, **P < .01, ***P < .001 and ****P < .0001. E and F, Images of spinal cord sections obtained from rabbits injected with AAV‐mRFP‐GFP‐LC3. The arrowheads indicate red dots. The arrows indicate yellow dots. Scale bar = 100 μm (10×). Scale bar = 10 μm (40×). The autophagic flux (red/yellow dot ratio per cell) in neuronal cells was quantified. The data are presented as the means ± SDs, n = 5. One‐way ANOVA followed by Tukey's multiple comparisons test. ****P < .0001

To verify the regulatory role of decompression in autophagy, the ratio of LC3 II/I and the expression of LC3 II and SQSTM1/p62 proteins were evaluated by WB (Figure 3A). The results showed that the LC3 II expression and the ratio of LC3 II/I were significantly increased in the spinal cord tissue of SCI rabbits compared with those in the Sham group (*P* < .0001 vs *P* < .0001), which indicated that LC3 II accumulated after injury. Compared with the SCI group, the LC3 II expression and the ratio of LC3 II/I were significantly decreased after decompression (in both the Durotomy and Piotomy groups), with the most significant decrease in the Piotomy group (*P* < .0001 vs *P* < .0001), which indicated that the LC3 II that accumulated due to damage was consumed after decompression. Compared with the Piotomy + CQ group, the ratio of LC3 II/I was significantly decreased in the Piotomy group and CQ group (*P* < .0001 vs *P* = .0005). Compared with the Piotomy + CQ group, LC3 II protein expression was significantly decreased in the Piotomy group and CQ group (*P* < .0001 vs *P* = .0131), which indicated that the degradation and generation of LC3 II protein were both promoted after piotomy. p62 was significantly increased in the spinal cord tissue of SCI rabbits compared with that in the Sham group (*P* = .0008), which indicated that the degradation of autophagy was impaired after injury. Compared with the SCI group, no significant differences in p62 protein were observed in the CQ group (*P* = .8473), indicating dysfunction of autophagic substrate degradation after SCI. Compared with the SCI group, p62 protein expression was significantly decreased after decompression (both in the Durotomy and Piotomy groups), with the most significant decrease in the Piotomy group (*P* < .0001), which indicated that the degradation of autophagy was enhanced after decompression. Compared with the Piotomy + CQ group, p62 protein expression was significantly decreased in the Piotomy group (*P* < .0001), which indicated that CQ inhibited the degradation of p62 protein and that the decreased p62 expression after decompression was at least partially caused by the autophagy pathway (Figure 3B‐D). Moreover, autophagic flux was observed to examine the effect of decompression on autophagy. Compared with the Sham group, the autophagic flux was significantly decreased in the SCI group (*P* < .0001). Compared with the SCI group, the autophagic flux was significantly increased after decompression (Durotomy and Piotomy groups) (*P* < .0001 vs *P* < .0001). The autophagic flux in the Piotomy group was higher than that in the Durotomy group (*P* < .0001). However, compared with the Piotomy group, the autophagic flux was significantly decreased after CQ administration (*P* < .0001) (Figure 3E,F). These results suggest that decompression increased autophagic flux, whereas CQ impaired autophagic flux.

### 3.4 Administration of CQ partially reversed the apoptosis inhibition caused by decompression after SCI

**Figure 4:**
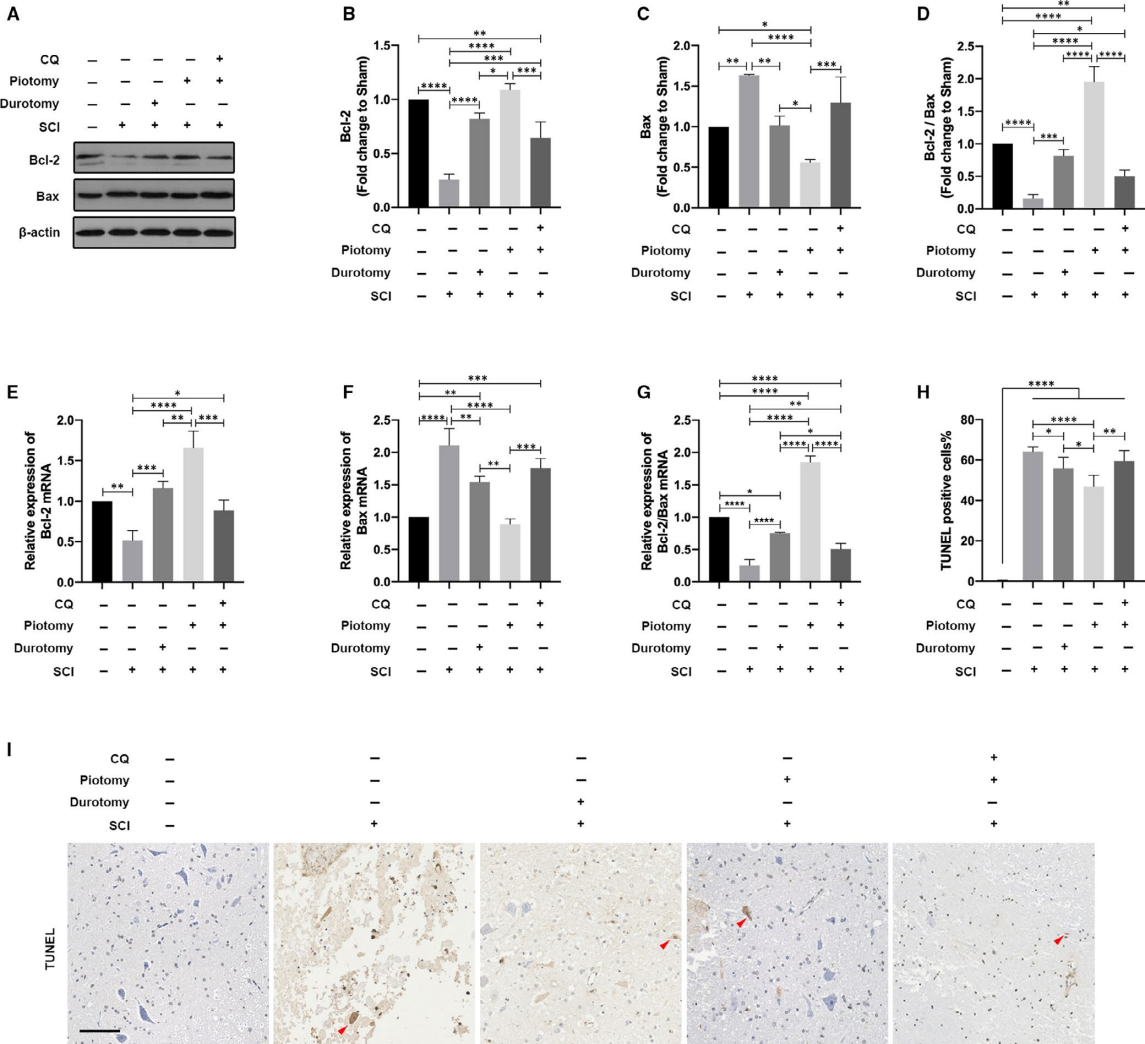
Administration of CQ partially reversed apoptosis alleviation caused by decompression after SCI. A‐D, Western blotting and quantitative analysis of changes in Bcl‐2 and Bax expression in rabbits. The data are presented as the means ± SDs, n = 3. One‐way ANOVA followed by Tukey's multiple comparisons test. *P < .05, **P < .01, ***P < .001 and ****P < .0001. E‐G, PCR of the levels of Bcl‐2 and Bax mRNA in rabbits. The data are presented as the means ± SDs, n = 3. One‐way ANOVA followed by Tukey's multiple comparisons test. *P < .05, **P < .01, ***P < .001 and ****P < .0001. H and I, Representative images and the quantitative analysis of spinal cord transverse sections stained with TUNEL in rabbits. The red arrows indicate TUNEL‐positive cells. Scale bar = 100 μm. The TUNEL‐positive cell percentage in the ventral horn of the spinal cord was quantified. n = 5. One‐way ANOVA followed by Tukey's multiple comparisons test. *P < .05, **P < .01 and ****P < .0001

To investigate the effect of decompression on apoptosis after SCI, we examined the protein expression and mRNA levels of Bax and Bcl‐2 at 72 hours after injury in the Sham, SCI, Durotomy, Piotomy and Piotomy + CQ groups. Compared with the Sham group, Bcl‐2 protein expression and mRNA levels were decreased, the ratio of Bcl‐2/Bax protein and mRNA levels were decreased, and Bax protein expression and mRNA levels were increased in the SCI group. The Durotomy and Piotomy groups exhibited significantly higher Bcl‐2 protein expression and mRNA levels, higher ratios of Bcl‐2/Bax protein and mRNA and lower Bax protein expression and mRNA levels than the SCI group. The Piotomy group exhibited significantly higher Bcl‐2 protein expression and mRNA levels, higher ratios of Bcl‐2/Bax protein and mRNA levels and lower Bax protein expression and mRNA levels than the Durotomy group. However, compared with the Piotomy group, Bcl‐2 protein expression and mRNA levels were significantly decreased (*P* = .0002 vs *P* = .0002), the ratio of Bcl‐2/Bax protein and mRNA was significantly decreased (*P* < .0001 vs *P* < .0001), and Bax protein expression and mRNA levels were significantly increased (*P* = .0010 vs *P* < .0002) after CQ administration (Figure 4A‐G).

Moreover, the number of TUNEL‐positive cells in the ventral horn of the spinal cord was observed. The number of TUNEL‐positive cells was significantly higher in the SCI group than in the Sham group (*P* < .0001). Compared with the SCI group, the number of positive cells was significantly decreased in the Durotomy and Piotomy groups, with the most significant decrease in the Piotomy group (Durotomy: *P* = .0496; Piotomy: *P* < .0001). The Piotomy group showed significantly fewer TUNEL‐positive cells than the Durotomy group (*P* = .0243). However, compared with the Piotomy group, the number of positive cells was significantly increased after CQ administration (*P* = .0014) (Figure 4H,I). These results suggested that apoptosis is inhibited by decompression but aggravated after CQ administration.

### 3.5 Administration of CQ partially reversed structural damage alleviation caused by decompression after SCI

**Figure 5:**
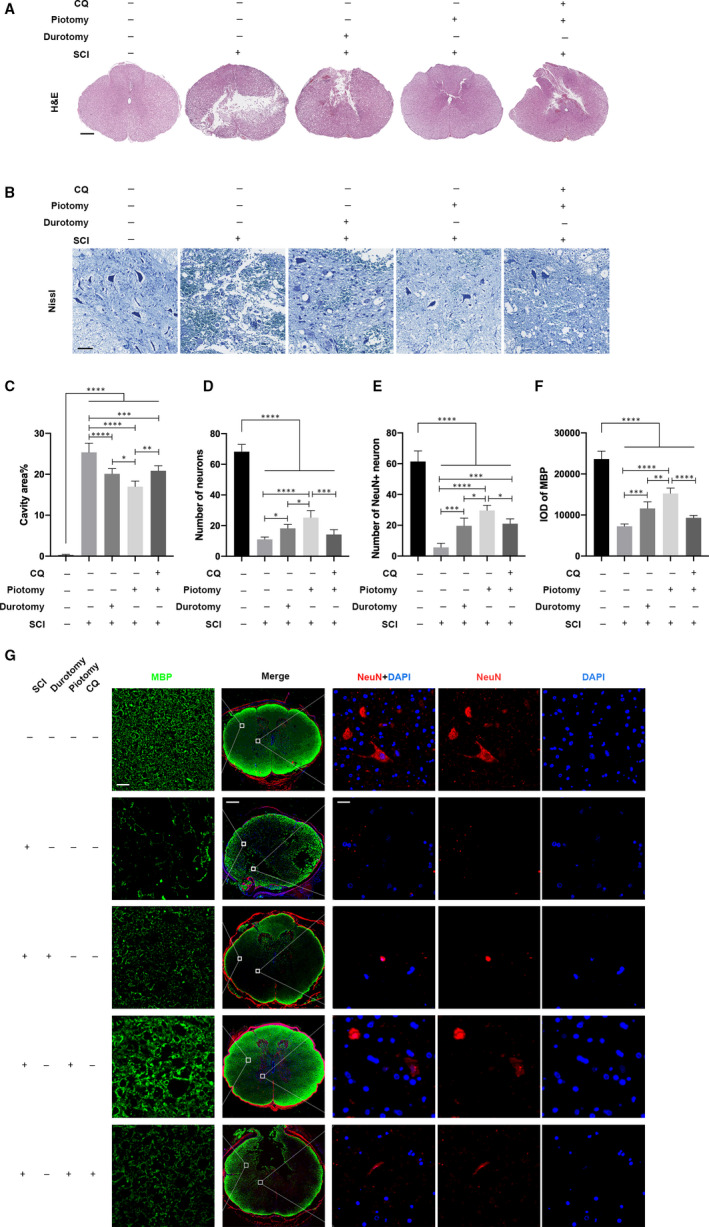
Administration of CQ partially reversed the structural damage alleviation caused by decompression after SCI. A and C, Representative images and the quantitative analysis of spinal cord transverse sections stained with H&E in rabbits. Scale bar = 500 μm. The cavity area% in the spinal cord sections was quantified. n = 5. One‐way ANOVA followed by Tukey's multiple comparisons test. *P < .05, **P < .01, ***P < .001 and ****P < .0001. B and D, Representative images and the quantitative analysis of spinal cord transverse sections stained with Nissl in rabbits. Scale bar = 100 μm. The numbers of neurons in the ventral horn of the spinal cord were quantified. n = 5. One‐way ANOVA followed by Tukey's multiple comparisons test. *P < .05, ***P < .001 and ****P < .0001. E‐G, Representative images and the quantitative analysis of spinal cord transverse sections stained with anti‐NeuN and anti‐MBP in rabbits. Scale bars = 20, 500 and 20 μm (from left to right). The IOD of MBP and the number of NeuN^+^ neurons in the spinal cord sections were quantified. n = 5. One‐way ANOVA followed by Tukey's multiple comparisons test. *P < .05, **P < .01, ***P < .001 and ****P < .0001

The difference in the morphology of tissues from the Sham, SCI, Durotomy, Piotomy and Piotomy + CQ groups was analysed by H&E staining 72 hours after injury (Figure 5A). The ratio of the cavity area to the spinal cord cross‐sectional area by H&E staining was calculated to histologically show the function of the spinal cord. Compared with the Sham group, severe damage to the white matter and grey matter was observed after SCI. In contrast, compared with the SCI group, obvious therapeutic effects were observed after decompression, with a decreased cavity area ratio in the white matter and grey matter (*P* < .0001). Compared with the Durotomy group, the Piotomy group showed a significantly decreased cavity area ratio (*P* = .0166). However, compared with the Piotomy group, the ratio of the cavity area was significantly increased after CQ administration (*P* = .0028) (Figure 5C). Neurons stained with Nissl were counted to examine the effect of decompression on the loss of neurons in the ventral horn (Figure 5B). Compared with the SCI group, increased neurons were observed after decompression, with the most significant increase in the Piotomy group (Durotomy: *P* = .0316, Piotomy: *P* < .0001) (Figure 5D). Moreover, neurons stained with NeuN in the ventral horn and the IOD of MBP were observed (Figure 5G). Decompression significantly reversed the decrease in NeuN‐positive neurons caused by SCI (Durotomy: *P* = .0007, Piotomy: *P* < .0001). However, compared with that in the Piotomy group, the number of NeuN‐positive neurons was significantly decreased after CQ administration (*P* = .0470) (Figure 5E). Compared with the SCI group, the IOD of MBP was significantly increased after decompression (Durotomy: *P* = .0003; Piotomy: *P* < .0001). The Piotomy group showed a significantly higher IOD of MBP than the Durotomy group (*P* = .0025). However, compared with that in the Piotomy group, the IOD of MBP was significantly decreased after CQ administration (*P* < .0001) (Figure 5F). Taken together, these results demonstrated that decompression alleviates the structural damage caused by SCI, whereas the structural damage is aggravated after CQ administration.

### 3.6 Decompression affects the function of lysosomes after SCI

**Figure 6:**
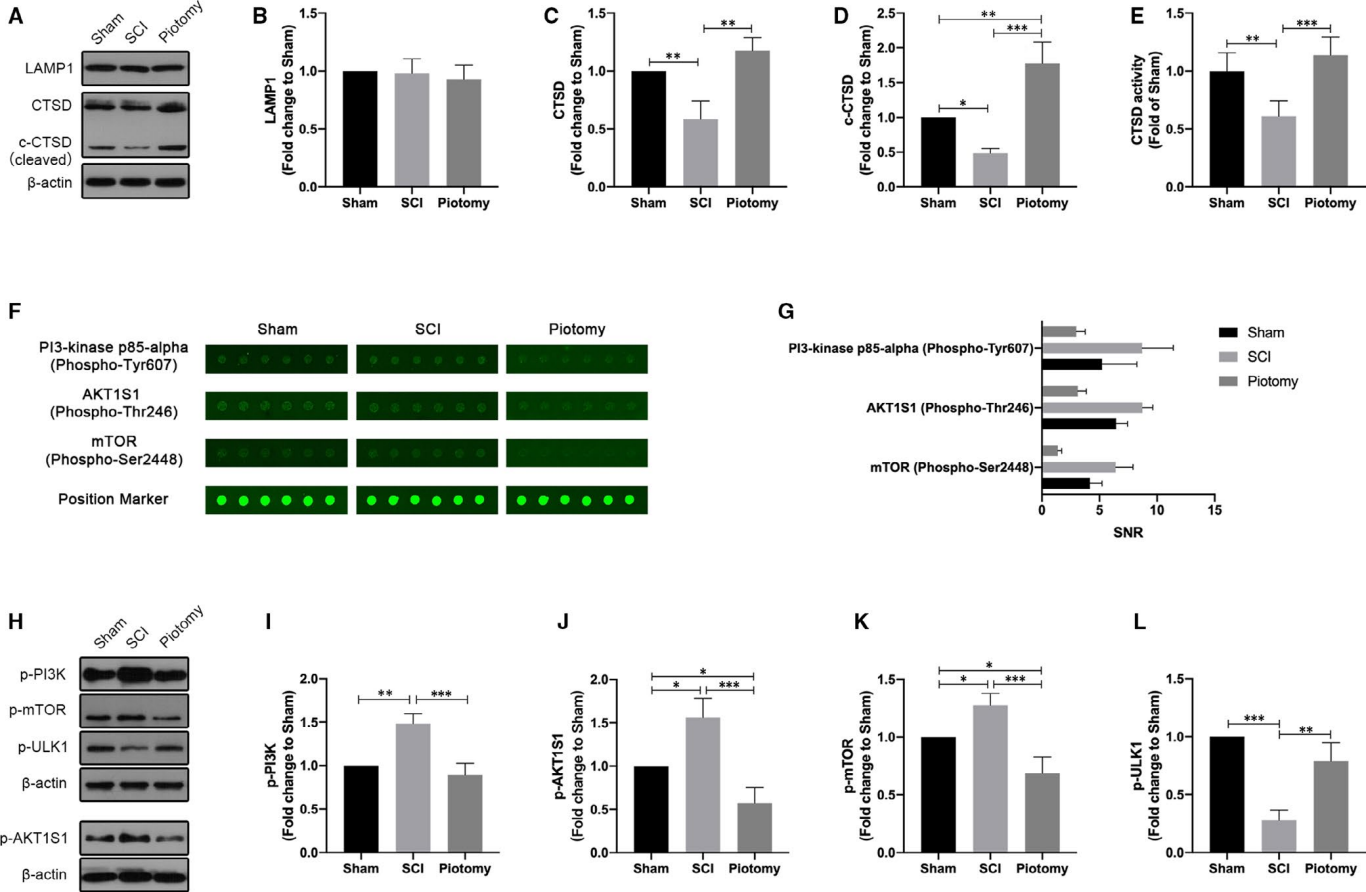
Decompression may affect the function of lysosomal and mTOR pathway after SCI. A‐D, Western blotting and quantitative analysis of changes in LAMP1 and CTSD expression in rabbits. The spinal cord tissue was fractionated to isolate lysosome‐enriched fraction and then processed for Western blot. Both full‐length precursor and cleaved CTSD are indicated. n = 3. One‐way ANOVA followed by Tukey's multiple comparisons test. *P < .05, **P < .01 and ***P < .001. E, The activity of the lysosomal enzyme CTSD was assessed. One‐way ANOVA followed by Tukey's multiple comparisons test. **P < .01 and ***P < .001. F and G, Images of the antibody array and the quantitative analysis of the signal‐to‐noise ratio (SNR) of each phosphor‐protein. See Table S1 and Figure S1 for full results and additional annotations. H‐L, Western blotting and quantitative analysis of changes in p‐PI3K, p‐AKT, p‐mTOR and p‐ULK1 expression in rabbits. n = 3. One‐way ANOVA followed by Tukey's multiple comparisons test. *P < .05, **P < .01 and ***P < .001

To determine the mechanisms by which IMP affects autophagic flux, we assessed lysosomal function in the Sham, SCI and Piotomy groups (Figure 6A‐E). Compared with the Sham group, the levels of precursor and cleaved CTSD protein in the lysosomes were decreased after SCI, and the enzymatic activity assay confirmed decreased CTSD activity, indicating that lysosomal function may be decreased due to injury. Compared with the SCI group, the precursor and cleaved CTSD protein levels and CTSD activity were increased in the Piotomy group, indicating that lysosomal dysfunction was relieved after decompression. Compared with the Sham group, no significant differences in LAMP1 were observed in the SCI and Piotomy groups, indicating that the size of the lysosomal compartment was not altered at 72 hours.

### 3.7 Decompression reverses the up‐regulated abundance of p‐PI3K, p‐AKT and p‐mTOR caused by SCI

To select the signalling pathways for decompression regulation, the spinal cord tissues of the Sham, SCI and Piotomy groups were analysed by antibody microarray (Figure 6F). The SNRs of p‐PI3K, p‐AKT and p‐mTOR were increased significantly in the SCI group compared with the Sham group. In contrast, the SNRs of p‐PI3K, p‐AKT and p‐mTOR in the Piotomy group were less than those in the SCI group (Figure 6G). See Table S1 and Figure S1 for full results and additional annotations. There were no ULK1 and p‐ULK1 sites in the antibody microarray. The expression of p‐PI3K, p‐AKT, p‐mTOR and p‐ULK1 was detected in spinal cord tissues from each group by WB 72 hours after SCI (Figure 6H‐L). p‐ULK1 was significantly decreased, and p‐PI3K, p‐AKT and p‐mTOR were significantly increased in spinal cord tissues from the SCI group compared with those in the Sham group. Decompression significantly up‐regulated p‐ULK1 and down‐regulated p‐PI3K, p‐AKT and p‐mTOR compared with the SCI group. These results suggested that decompression may activate the PI3K/AKT/mTOR signalling pathway after SCI.

## 4 DISCUSSION

In this study, we performed two methods of decompression (durotomy and piotomy) in a rabbit SCI model and observed autophagy and apoptosis of the injured spinal cord after decompression. We found that decreased IMP promotes the generation and degradation of LC3 II, promotes the degradation of p62 and enhances autophagic flux, thus alleviating apoptosis. Lysosomal dysfunction was reduced after decompression. Piotomy had better therapeutic effects on the histological recovery of the spinal cord tissue after SCI. However, the autophagy‐lysosomal pathway inhibitor CQ partially reversed apoptosis alleviation caused by piotomy after SCI, and the structural damage was also aggravated after CQ administration. Further analysis of the antibody microarray revealed that decompression reversed the up‐regulated abundance of p‐PI3K, p‐AKT and p‐mTOR caused by SCI.

After SCI, the intra‐ and extracellular water balance is blocked, cell oedema occurs and the extracellular space decreases. Extensive microvascular injuries and bleeding lead to haematoma formation. These factors lead to sharply increased mechanical stress in the spinal cord. The results of this study show that durotomy and piotomy effectively reduce IMP and alleviate apoptosis and structural damage after SCI. The spinal cord may still be bound by the pia mater and may not completely relieve the pressure increase caused by swelling of the spinal cord after durotomy. The spinal dura mater, arachnoid and pia mater of the spinal cord were incised by piotomy to reduce the pressure inside the spinal cord. Therefore, the decompression effect of piotomy was more obvious, which is similar to the findings of previous research.^25^ In this study, piotomy with lower pressure was more effective than durotomy. In the Piotomy group, autophagic flux was enhanced, apoptosis and demyelination were alleviated, and structural damage to the spinal cord was significantly alleviated.

Autophagy suppresses apoptotic cell death via a direct interaction between Beclin‐1 and the apoptosis regulator Bcl‐2.^26^, ^27^ Despite controversy, autophagy up‐regulation after SCI is thought to play a neuroprotective role^28^, ^29^, ^30^ that is related to microtubule stabilization, promoting axon regeneration and reducing the incidence of apoptosis in recovery.^18^, ^31^, ^32^ In this study, decompression simultaneously promoted LC3 formation and degradation. The accumulated LC3 caused by injury was consumed after decompression, resulting in significantly lower LC3 levels than in the SCI group. After decompression, p62 protein expression decreased, indicating increased autophagy degradation; an increased red/yellow dot ratio indicated increased autophagic flux. The apoptosis‐activating protein Bax decreased, Bcl‐2 protein increased, the Bcl‐2/Bax ratio increased, and the number of TUNEL‐positive cells decreased, indicating that apoptosis was suppressed after decompression. To further verify whether decompression after SCI regulates autophagy to inhibit apoptosis and reduce tissue damage, CQ was given as an autophagy‐lysosomal pathway inhibitor to inhibit autophagy after surgery in the Piotomy group.^33^ In the Piotomy + CQ group, the degradation of autophagy was inhibited, and autophagic flux was reduced. Compared with the Piotomy group, after CQ intervention, Bax protein increased, Bcl‐2 protein decreased, the Bcl‐2/Bax ratio decreased, the number of TUNEL‐positive cells increased and tissue damage also increased. These results indicate that administration of CQ partially reversed the apoptosis inhibition and structural damage alleviation caused by decompression after SCI. Therefore, decompression restores autophagic flux to alleviate apoptosis and structural damage to the spinal cord after SCI.

Considering that the degradation of autophagic substrates is conducted by lysosomes,^34^, ^35^ we evaluated the variation in lysosomal function after decompression to determine the mechanism by which IMP affects the autophagic flux. In this study, the precursor and cleaved CTSD protein levels decreased, and CTSD activity decreased, indicating that lysosomal function was impaired after SCI. Lysosomal dysfunction may lead to autophagic flux inhibition after SCI, which is consistent with the findings of previous studies.^36^, ^37^ Lysosomal dysfunction is reduced after decompression, which may be one of the reasons for the increased autophagic flux after surgical decompression after SCI.

In this study, we also explored the molecular mechanism by which decompression promotes recovery. Antibody microarray analysis showed that decompression reversed the up‐regulation of p‐PI3K, p‐AKT and p‐mTOR proteins caused by SCI. The PI3K/AKT/mTOR pathway, the most crucial upstream signalling pathway of autophagy, is well established as a regulator of a wide range of cellular processes that mediate cell survival and death, and this pathway is inhibited by malnutrition, hypoxia, external pressure or other stress conditions to suppress cell proliferation and enhance autophagy, eventually leading to cell death.^38^, ^39^ Akt is a member of the serine/threonine protein kinase family and is activated in a PI3K‐dependent manner in response to various stimuli.^40^ Phosphorylated class I PI3K and Akt activate mTOR.^41^ mTOR is a downstream target of the PI3K/AKT pathway.^42^ mTOR kinase is part of two mTOR complexes, mTORC1 and mTORC2, each of which has a different protein component.^43^ mTORC1 is a major negative regulator of autophagy.^44^ Inhibition of mTORC1 causes TFEB dephosphorylation and nuclear translocation, which subsequently increases lysosome biogenesis, and affects autophagic flux.^45^, ^46^, ^47^ Suppression of PI3K and Akt signalling inhibits mTOR phosphorylation at Ser2448, thereby inducing autophagy and increasing the levels of autophagy‐related proteins.^48^ Activation of this pathway promotes cell growth, differentiation and survival while down‐regulating apoptotic signals.^49^, ^50^ The ULK1 protein kinase is a key regulator of autophagy initiation and progression.^51^ In vitro, the mechanical induction of autophagy is TOR independent.^16^ However, there may be differences between in vivo and in vitro studies. In this study, there were significant differences in the expression of p‐PI3K, p‐AKT, p‐mTOR and p‐ULK1 protein in the rabbit SCI model before and after decompression. Decompression may regulate autophagy by activating the PI3K/AKT/mTOR signalling pathway.

Most current studies support the importance of early decompression after SCI,^11^, ^52^ and pathological changes in the acute stage may have a significant impact on the functional prognosis of patients. In addition, considering that neuronal apoptosis incidence peaked 72 hours after SCI,^53^, ^54^, ^55^ and because defects in autophagy and lysosomal function may occur very early after SCI,^36^ we focused our analysis on the 72 hours after injury. Compared with SCI group, a recovery trend in motor function in rabbits was observed after decompression (both in the Durotomy and Piotomy groups), but there were no significant differences. An extended observation time may have yielded different results.

Due to the complexity of in vivo studies, it may be difficult to isolate mechanical signals and determine their individual effects. However, these results may provide a new molecular mechanism for the neuroprotective effect of decompression and the mechanisms of the effects of mechanical stress on autophagy after SCI. In addition, we cannot exclude the possibility that decompression stimulates autophagy through other potential signalling pathways after SCI. Therefore, further research is still needed. IMP after SCI may be an important factor in regulating autophagic flux. On the other hand, in addition to the direct regulation of mechanical stress, nutritional deficiencies and hypoxia are also effective stimuli for autophagy.^56^ Decompression increases blood flow to the spinal cord and rebuilds cerebrospinal fluid circulation, thus preventing the spinal cord from continuing ischaemic injury.^57^, ^58^ Decompression partially alleviated nutritional deficiencies and hypoxia in nervous tissue. Therefore, IMP may also have an indirect effect on autophagic flux.

## 5 CONCLUSIONS

Decompression promotes the degradation and generation of LC3 II and the degradation of p62 and restores autophagic flux to alleviate apoptosis and promote histological recovery after SCI. Lysosomal dysfunction was reduce after decompression. The therapeutic effects of piotomy may be better than those of durotomy. Decompression may reverse the up‐regulated abundance of p‐PI3K, p‐AKT and p‐mTOR caused by SCI.

## CONFLICT OF INTEREST

The authors confirm that there are no conflicts of interest.

## AUTHOR CONTRIBUTIONS

**Xin Zhang:** Conceptualization (equal); Data curation (lead); Formal analysis (lead); Investigation (lead); Methodology (lead); Project administration (lead); Writing‐original draft (lead). **Yingli Jing:** Data curation (equal); Formal analysis (equal); Investigation (equal); Methodology (equal); Project administration (equal); Writing‐original draft (equal). **Chuan Qin:** Data curation (equal); Formal analysis (equal); Investigation (equal); Methodology (equal); Writing‐original draft (equal). **Changbin Liu:** Data curation (equal); Formal analysis (equal); Investigation (equal); Methodology (equal); Project administration (equal). **Degang Yang:** Data curation (equal); Formal analysis (equal); Investigation (equal); Methodology (equal); Writing‐original draft (equal). **Feng Gao:** Data curation (equal); Investigation (equal); Project administration (equal); Resources (equal); Writing‐review & editing (equal). **Mingliang Yang:** Data curation (equal); Methodology (equal); Project administration (equal); Supervision (equal); Writing‐review & editing (equal). **Liangjie Du:** Data curation (equal); Methodology (equal); Project administration (equal); Supervision (equal); Writing‐review & editing (equal). **Jianjun Li:** Conceptualization (lead); Data curation (supporting); Formal analysis (supporting); Funding acquisition (lead); Investigation (supporting); Methodology (equal); Project administration (lead); Supervision (lead); Writing‐original draft (supporting); Writing‐review & editing (supporting).

## Supporting information

Appendix S1

Table S1

Figure S1

## Data Availability

All data generated or analysed during this study are included in this published article.
