# Supplementary material for: Mechanical stress regulates autophagic flux to affect apoptosis after spinal cord injury
Source: J Cell Mol Med. 2020 Sep 17;24(21):12765–76. doi: 10.1111/jcmm.15863 (PMC7686991; doi:10.1111/jcmm.15863)
Supplement: Supplementary file 1 — Appendix S1 [file JCMM-24-12765-s001.docx]

**Appendix S1. Materials and Methods**

***Sampling***

The spinal cord was removed immediately after each animal was sacrificed by anesthesia at 72 hours after injury. A spinal cord segment with a length of 5 mm was taken from around the point of injury (T10), fixed in 4% paraformaldehyde for 24 hours and stored for future use. The spinal cord segments in the rest of the rabbits within the group were removed, rapidly frozen in liquid nitrogen, and then stored in a refrigerator at −80°C for future use.

***TUNEL***

TUNEL staining was used to detect apoptosis of spinal cord anterior horn neurons. To block endogenous peroxidase activity, sections were incubated with a methanol solution containing 0.2% H_2_O_2_ for 0.5 hours. Then, the sections were treated with a TUNEL reaction mixture (Millipore, Shanghai, China), and the sections were incubated at 37°C for 60 min. After HistoFAXS 3.0 scans of all tissue section images, the total number of cells in the anterior horn of the spinal cord and the number of TUNEL-positive cells were manually counted. Apoptosis index = (TUNEL-positive cells)/(total cells) × 100%.

***Real-time PCR***

The total RNA of the collected spinal cord was extracted using an RNA extraction reagent (G3013, Servicebio, Wuhan, China) according to the manufacturer’s instructions. First-strand cDNAs were synthesized using a RevertAid First Strand cDNA Synthesis Kit (#K1622, Thermo Scientific Fermentas, Vilnius, Lithuania). qPCR was performed on a real-time PCR system (StepOne Plus, Applied Biosystems ABI, CA, USA). Gene expression was expressed as the mRNA level, which was normalized to the mRNA level of a standard housekeeping gene (β-actin) using the ΔΔCT method. Three independent experiments were performed for each set of PCR analyses. The primers used for qPCR were as follows: Bax, F, 5′-CGACGGCAACTTCAACTGGG-3′, R, 5′-TTCTTCCAGATGGTGAGTGAGGC-3′; Bcl-2, F, 5′-GTGGCCTTCTTTGAGTTCGG-3′, R, 5′-GAGGGTGATGCAAGCTCCTAT-3′; and β-actin, F, 5′-CGCAGAAACGAGACGAGATTG-3′, R, 5′-GATGCTCGCTCCAACGACTG-3′.

***Autophagic flux measurement***

The rabbits were injected intrathecally with AAV-mRFP-GFP-LC3 (purchased from Hanbio, Shanghai, China) at the rostral segment of the injury center (T9) two weeks before SCI for subsequent autophagic flux measurement. Using a stereotactic device (51750, Stoelting, Wood Dale, USA) and a microinjection pump (53311, Stoelting) equipped with a 25-μL microinjector (Hamilton, Reno, NV, USA) and a 33-G needle, the animals were internally injected with 20 µL of construct at a speed of 1 μL/min. At 72 hours after SCI, the spinal cord was removed by the above method and then dehydrated and embedded in paraffin. For each group, n = 5, and each sample was cut into coronal sections (4 μm) at the center of the injury. After the images were scanned with Pannoramic 250, the two channels were merged for colocalization analysis (i.e., red and yellow dot analysis), and the red and yellow dots in neuronal cells (definition: intact cells, nuclei and axons) were manually counted in a double-blind manner to determine the difference in autophagic flux between groups.

***Subcellular fractionation***

Approximately 5-mm fragments of spinal cord tissue centered on the injury site in rabbits were collected from the Sham group and at 72 hours after SCI/decompression; they were homogenized in ice-cold buffered solution containing 0.32 M sucrose, 10 mM HEPES and protease and phosphatase inhibitors. Homogenates were centrifuged at 800 g for 10 min at 4°C to spin down the nuclei. Supernatants were sequentially centrifuged at 20000 g for 20 min at 4°C to spin down the heavy membrane/crude lysosomal fractions and at 100000 g for 1 hour at 4°C to spin down the light membrane fractions.^1^ All pellets were resuspended in homogenization buffer, and the protein concentration was estimated using BCA reagent (Thermo Fisher Scientific). Fractions were then analyzed by WB (n = 3).

***Lysosomal activity assays***

The CTSD assay was performed using a fluorometric assay kit (ab65302, Abcam, Cambridge, UK) according to the manufacturers’ instructions. Briefly, approximately 5 mm of spinal cord tissue centered on site of injury was collected from rabbits and homogenized in the ice-cold cell lysis buffer provided in the kit. Homogenates were centrifuged at 15,000 g for 5 min at 4°C. The protein concentration was estimated by the BCA method. A total of 50 ng of protein were used per assay. Fluorescence released from the synthetic substrate was measured using a multimode plate reader (EnSpire, Perkin Elmer Singapore Pte Ltd.) at Ex/Em = 328/460 nm to assess CTSD activity.

***Antibody microarray analysis***

Antibody microarray analysis was commissioned by Wanen Biotechnologies Inc. (Shanghai, China). Spinal cord tissues were extracted at 72 hours after surgery and used for Phospho Explorer antibody array (CSP100 Plus, Full Moon BioSystems, CA, USA). The antibody microarray binds 304 highly specific antibodies, including 157 phosphorylated antibodies and 144 nonphosphorylated antibodies. There were 6 replicates per antibody. For the obtained images, GenePix Pro v6.0 (Axon Instruments, Foster, CA, USA) software was used to read the raw data. The signal-to-noise ratio (SNR) of the background, positive and internal control sites was counted. The internal reference was Tubulin-b. The KEGG database was used to further analyze key signaling pathways.

***Statistical analysis***

GraphPad Prism 8.0 (GraphPad Software, Inc) was used for statistical analysis. The number of animals in all studies was determined by power analysis (power of 0.8 with an alpha value of 0.05). Two-way repeated measures analysis of variance (ANOVA) followed by Tukey's multiple comparisons test were used to compare the differences in IMP at different time points. One-way ANOVA followed by Tukey's multiple comparisons test were used to compare the differences between the Sham, SCI, Durotomy, Piotomy, Piotomy+CQ and CQ groups. P <0.05 was considered statistically significant.

**References**

1. Sarkar C, Zhao Z, Aungst S, Sabirzhanov B, Faden AI, Lipinski MM. Impaired autophagy flux is associated with neuronal cell death after traumatic brain injury. *Autophagy* 2014;10:2208-2222.
