# Supplementary figures and images for: Mechanical stress regulates autophagic flux to affect apoptosis after spinal cord injury
Source: J Cell Mol Med. 2020 Sep 17;24(21):12765–76. doi: 10.1111/jcmm.15863 (PMC7686991; doi:10.1111/jcmm.15863)

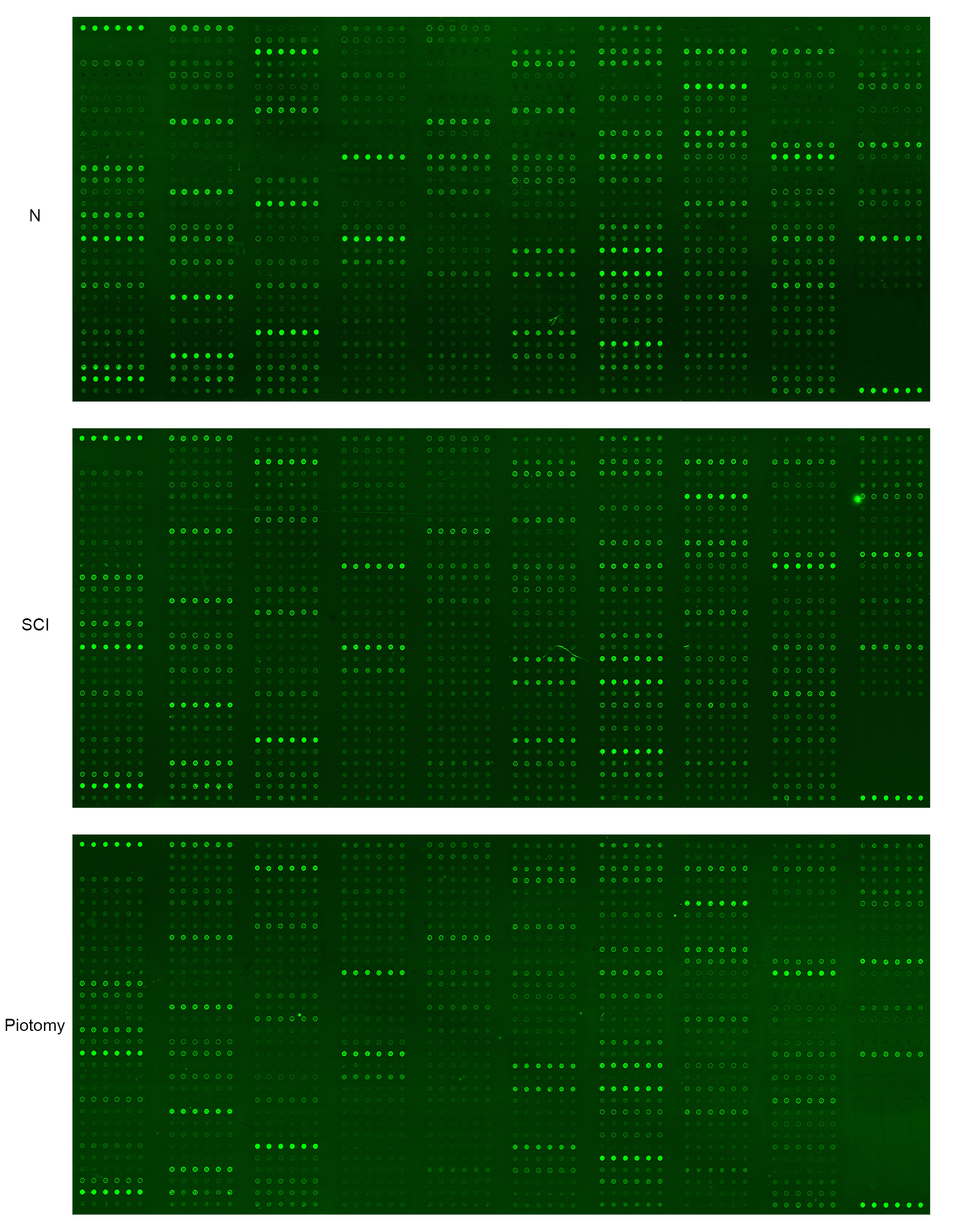

Supplement: Supplementary file 3 — Figure S1 [file JCMM-24-12765-s003.tif]
